# Preparation and Application of Sodium Alginate-Based Composite Hydrogels in Wound Dressings

**DOI:** 10.3390/gels12060458

**Published:** 2026-05-24

**Authors:** Dandan Chen, Yan He, Xinyue Zhang, Longyi Nan, Xin Jin, Yan Zheng, Chao Sun, Jianpeng Guo, Xinyu Li

**Affiliations:** 1 Key Laboratory of Natural Medicines of the Changbai Mountain, Ministry of Education, College of Pharmacy, Yanbian University, Yanji 133002, China; 2025010974@ybu.edu.cn (D.C.); 0000008943@ybu.edu.cn (X.Z.); nanlongyi2018@163.com (L.N.); 0000007947@ybu.edu.cn (X.J.); zhengyan_0308@163.com (Y.Z.); 2 Jilin Collaborative Innovation Center for Antibody Engineering, Jilin Medical University, Jilin 132013, China; hy0725@jlmu.edu.cn; 3 Department of Physics, Jilin University, Changchun 130012, China; sunc344@jlu.edu.cn; 4 Department of Polymer Materials & Engineering, College of Engineering, Yanbian University, Yanji 133002, China

**Keywords:** wound dressing, hemostasis, wound healing, sodium alginate composite hydrogel, antibacterial

## Abstract

Wound healing is a complex physiological process involving multiple stages, including hemostasis, inflammation, proliferation, and remodeling, which imposes high demands on the functionality and adaptability of wound repair materials. Hydrogels, as a class of novel materials, have become ideal wound dressings due to their excellent biocompatibility, breathability, and conformability. Sodium alginate-based composite hydrogels offer advantages such as readily available raw materials and mild preparation conditions. They can also endow materials with properties including antibacterial, anti-inflammatory, hemostatic, and pro-angiogenic effects, meeting the application requirements for multifunctional and highly efficient wound dressings. As a result, they have attracted considerable attention in the field of wound repair. This article introduces the preparation methods of physically and chemically crosslinked sodium alginate-based composite hydrogels, as well as the drug release mechanisms from these hydrogels. It elaborates on their applications in wound dressings, discusses key challenges including difficulties in large-scale preparation, high barriers to clinical translation, insufficient long-term in vivo stability, and low integration of intelligent functions, and outlines future research directions in terms of large-scale fabrication, regulatory compliance, long-term safety, and intelligent design. This review aims to provide a theoretical basis for the development of novel sodium alginate-based composite hydrogels for wound dressings and to promote their clinical translation and practical application in this field.

## 1. Introduction

Wound healing is a multi-stage physiological process that mainly consists of four phases: hemostasis, inflammation, cell proliferation, and tissue remodeling. These phases work synergistically to accomplish wound repair. An ideal wound dressing can provide a stable and moist environment for wound repair and significantly improve healing efficiency. Traditional wound dressings such as gauze, foam, and sponge generally suffer from drawbacks including poor moisture retention and limited absorption capacity. They tend to cause secondary damage and increased pain during dressing changes, making them unable to meet the current requirements for efficient repair in wound care. Therefore, the development of novel wound dressings with biosafety, high fluid absorption, non-adhesion to tissues, and multifunctional repair capabilities has become an important research direction in the field of wound repair [1].

Hydrogels are three-dimensional hydrophilic network structures formed by physical entanglement or chemical crosslinking of polymer chains. Owing to their high water content and excellent mechanical flexibility, hydrogels have emerged as highly promising materials for wound dressings. Nevertheless, conventional hydrogels generally suffer from poor gelation performance, weak mechanical strength, and single function, which limit their application in the repair of complex wounds. To address these bottlenecks, researchers commonly select appropriate matrix materials for composite modification to satisfy practical application requirements. Among various hydrogel matrix materials, sodium alginate possesses the advantages of abundant sources and favorable gelation ability, making it a natural polymer material with great application potential. However, pure sodium alginate hydrogels exhibit poor mechanical strength, low structural stability, and limited functionality. Therefore, using sodium alginate as the matrix to incorporate multiple components and construct multifunctional composite hydrogels has become an important approach for optimizing material properties, adapting to complex wound repair, and promoting their engineering applications in biomedicine. In recent years, sodium alginate-based composite hydrogels have achieved rapid development in the field of wound dressings. Nevertheless, systematic reviews focusing on their preparation strategies, drug release mechanisms and multifunctional repair applications are still relatively scarce [2].

Based on the above research background, this paper systematically reviews the research progress of sodium alginate-based composite hydrogels in the field of wound dressings, aiming to construct a complete logical chain from material preparation and drug release mechanisms to final biomedical applications. The article first elaborates on physical and chemical crosslinking methods and clarifies their effects on the structure and properties of hydrogels. It then thoroughly discusses drug release mechanisms including sustained release, controlled release, and stimuli-responsive release, revealing the intrinsic relationships between these mechanisms and the material structure and components. Finally, it focuses on the specific applications of these hydrogels in wound repair, such as antibacterial, anti-inflammatory, hemostatic, and pro-angiogenic effects. On this basis, the existing problems in current research and the challenges in clinical translation are analyzed, and the future development directions of large-scale, precise, and multifunctional sodium alginate wound repair materials are prospected. This review aims to provide systematic theoretical references and scientific basis for the rational design and development of high-performance wound dressings.

## 2. Preparation of Sodium Alginate-Based Composite Hydrogels

Sodium alginate, as a natural polysaccharide, possesses excellent biocompatibility, water absorption, and hemostatic properties. It can form three-dimensional network hydrogels through crosslinking reactions, making it an ideal substrate for wound dressings that can effectively absorb wound exudate and maintain a moist healing environment. However, single-component sodium alginate hydrogels suffer from insufficient mechanical strength and limited functionality, which makes them inadequate for complex wound applications in areas such as joints and other moving parts. Therefore, sodium alginate is often combined with other components such as polyvinyl alcohol, gelatin, and plant extracts to synergistically enhance the mechanical properties, antibacterial activity, and healing-promoting ability of the hydrogels. The preparation of sodium alginate-based composite hydrogels primarily relies on two types of techniques: physical crosslinking and chemical crosslinking. Both methods enable the controllable fabrication of gel structure and performance, providing diverse options for the development and clinical application of wound dressings. These preparation methods not only determine the physicochemical properties of hydrogels, but also lay the foundation for subsequent drug encapsulation and release, directly influencing their biological functions in wound repair.

### 2.1. Physical Cross-Linking

Physical crosslinking refers to a method in which different polymer chains form a complex crosslinked network through mechanisms such as chain entanglement, hydrogen bonding, van der Waals forces, hydrophobic interactions, and electrostatic attraction. Unlike chemical crosslinking, physical crosslinking is a milder process with controllable conditions and does not require the addition of toxic crosslinking agents. Therefore, the resulting hydrogels exhibit higher biosafety and environmental responsiveness. Because these interactions are reversible weak bonds, the structure of hydrogels formed by physical crosslinking can dynamically adjust in response to changes in external environmental factors such as temperature, pH, and ionic strength, endowing them with good adaptability and degradability in biomedical fields like wound dressings and drug delivery. In summary, physical crosslinking methods, including ionic crosslinking, hydrogen bonding crosslinking, hydrophobic interaction and electrostatic interaction, occupy an important position in the preparation of sodium alginate-based composite hydrogels due to their advantages such as simple operation, mild preparation conditions and no need for toxic crosslinkers. These methods endow hydrogels with excellent biocompatibility, pH responsiveness, self-healing ability and injectability, enabling them to have broad application prospects in wound dressings and drug delivery fields. Nevertheless, physical crosslinking is mainly limited by weak ionic bond force, poor structural stability and easy dissociation under environmental influences. Therefore, in clinical transformation, while taking advantage of its superior biosafety and dynamic responsiveness, it is necessary to balance its relatively low mechanical strength and stability. Especially in application scenarios requiring long-term stable support or bearing large mechanical stress, it is essential to combine it with other crosslinking strategies to compensate for its deficiencies. Table 1 summarizes the four most commonly used crosslinking methods for preparing sodium alginate-based composite hydrogels, along with their respective advantages and disadvantages [3].

#### 2.1.1. Ionic Crosslinking

**Figure 1:**
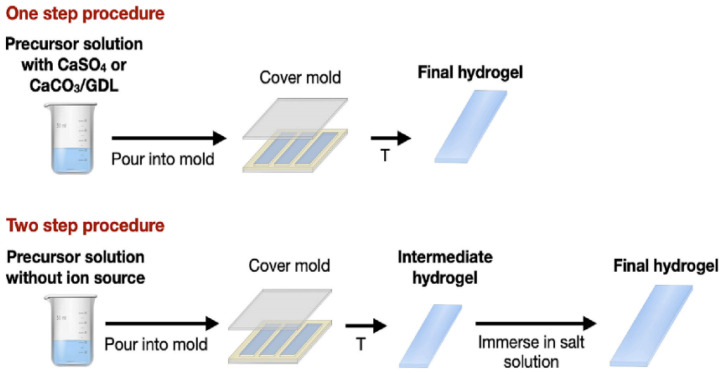
Schematic diagram of the preparation of polyacrylamide/sodium alginate double-network hybrid hydrogels by one-step method and two-step method [5]. Copyright 2023, Elsevier.

Ionic crosslinking offers the advantages of simple operation and rapid gel formation, making it one of the most commonly used methods for preparing sodium alginate-based composite hydrogels. Its core mechanism involves the specific binding of metal ions to the carboxyl groups of four guluronic acid (G) units within the sodium alginate molecular chains, forming a three-dimensional “egg-box” network structure [20,21]. To further enhance the structural stability and mechanical properties of sodium alginate-based composite hydrogels, current research often employs ionic crosslinking in synergy with other interactions or optimizes the crosslinking conditions. Zhou et al. [4] successfully prepared sodium alginate-polyacrylamide (SA-PAM) hydrogels with a double-network structure by introducing divalent ions (Cu^2+^, Zn^2+^, Sr^2+^, Ca^2+^). In this process, acrylamide polymerizes in the presence of a crosslinking agent to form a covalently crosslinked polyacrylamide and sodium alginate molecular chain network. Subsequently, this network is immersed in a divalent cation chloride solution, where the divalent ions form ionic bonds with the G-blocks of the alginate chains, thereby achieving ionic crosslinking. Houben et al. [5] formed a crosslinked network by combining multivalent cations (such as Ca^2+^, Fe^3+^, Al^3+^, Ba^2+^, Sr^2+^) with the carboxylic acid groups of alginate, as shown in Figure 1. This study compared various calcium-based crosslinking strategies and found that the CaCO_3_/GDL one-step method produced gels with a more homogeneous structure and superior mechanical properties, making it more suitable for 3D printing and injection molding. The introduction of different ions (e.g., Ba^2+^, Fe^3+^) can significantly modulate the mechanical strength of the gel; generally, the larger the ionic radius or the higher the valence state, the stronger the resulting gel. However, ionic crosslinked hydrogels may experience partial ion exchange and mild mechanical attenuation under physiological conditions. This limitation can be significantly mitigated via composite modification or crosslinking optimization, enabling satisfactory stability for short-term wound applications. Liu et al. [6] prepared a two-component hydrogel (AgBs-IG) consisting of a sol component and a spray component. The sol component (Ag-I) was primarily composed of sodium alginate (SA), polyvinyl alcohol (PVA), and itaconic acid (IA); the spray component (Bs-G) consisted of calcium chloride and gallic acid (GA). After filling the wound bed with the Ag-I sol and spraying Bs-G, instantaneous ionic crosslinking occurred between Ca^2+^ ions and the carboxyl groups of sodium alginate, rapidly forming a smooth, adhesive, in situ AgBs-IG hydrogel layer. Yamauchi et al. [7] partially substituted sodium ions in sodium alginate with calcium ions to synthesize calcium-crosslinked sodium alginate (Alg-Na/Ca). A series of samples were prepared by adjusting calcium content, molecular weight, mannuronic acid/guluronic acid (M/G) ratio and particle size, and their swelling ratio and bursting pressure were measured. The results showed that the materials swelled rapidly after contacting with normal saline, and the samples with calcium content ranging from 74.1% to 77.0% exhibited excellent swelling behavior and high bursting pressure within two minutes.

The most prominent advantages of the ionic crosslinking method lie in its mild process, simple operation, and excellent biocompatibility. It only requires the electrostatic complexation of multivalent metal ions (such as Ca^2+^, Zn^2+^, Ba^2+^, Fe^3+^, etc.) with the carboxyl groups of the G-blocks of sodium alginate via the “egg-box model,” enabling rapid gelation under aqueous conditions at room temperature without the need for toxic chemical crosslinking agents. It offers strong tunability; by varying the type, valence state, and introduction method of the ions, the gelation rate, mechanical strength, pore structure, and swelling behavior can be precisely controlled. Furthermore, specific metal ions can impart additional functionalities to the gel, such as the antibacterial activity and wound healing-promoting ability of zinc ions. However, insufficient stability in physiological environments remains its major bottleneck. For the repair of complex wounds requiring long-term stable support or bearing significant mechanical stress, the limitations of single ionic crosslinking are particularly pronounced. Future research should focus on exploring effective approaches to mitigate mechanical attenuation caused by ion exchange through composite modification or synergistic integration with other crosslinking strategies, while optimizing ion release kinetics, so as to achieve more precise biological functions and broader clinical applications.

#### 2.1.2. Hydrogen Bonding Crosslinking

**Figure 2:**
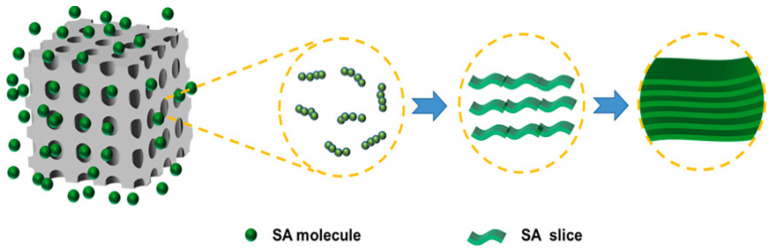
Mechanism diagram of the lamellar structure of sodium alginate/polyacrylamide (SA/PAM) hydrogels [8]. Copyright 2020, Elsevier.

Hydrogen bonding crosslinking refers to a crosslinking method for constructing hydrogels, in which the hydroxyl (-OH) or carboxyl (-COOH) groups in sodium alginate molecules interact with complementary groups of other molecules to form a dynamic hydrogen bonding network. Hydrogen bonds exhibit dynamic reversibility in sodium alginate-based hydrogel systems, allowing them to break and reform under stress, thereby endowing the hydrogels with self-healing capability. Furthermore, the stability of the hydrogen bonding network is sensitive to the pH of the system. Under acidic conditions, protonation of carboxyl groups enhances hydrogen bonding interactions, leading to a denser gel structure [22]. Leveraging the reversibility of hydrogen bonds, Zhao et al. [8] prepared a semi-interpenetrating polymer network (semi-IPN) hydrogel by promoting the self-assembly of sodium alginate (SA) within a porous polyacrylamide (PAM) matrix through dynamic hydrogen bonding, as shown in Figure 2. SA molecules induce and influence their own self-assembly within the porous PAM structure via intermolecular hydrogen bonding, forming ordered micro-lamellar structures. These micro-lamellae of SA continue to grow, forming thin SA layers. The SA layers further drive and induce PAM to complete the regulation of the semi-interpenetrating polymer network (semi-IPN structure). Liu et al. [9] developed a hydrogen bonding-driven hydrogel. Hydrogen bonding crosslinking endowed the hydrogel with several excellent properties, including high stretchability exceeding 700%, strong adhesion up to 7.5 kPa to various surfaces, remarkable toughness and resilience, good biocompatibility, and high fluid absorption capacity. Iliopoulou et al. [10] prepared an alginate-based composite hydrogel. Under acidic conditions, the protonated alginate backbone formed intermolecular hydrogen bonds with the poly(N-isopropylacrylamide) (PNIPAM) side chains, constructing a secondary physical crosslinking network. This not only allowed the hydrogel to maintain elasticity at room temperature and significantly enhance mechanical strength, but also achieved precise slowing of drug release by increasing the medium viscosity, while endowing the material with excellent self-healing and injectable properties. Cui et al. [11] prepared a hydrogel for skin wounds contaminated with radionuclides using polyacrylamide (PAM), sodium alginate (SA), and diethylenetriaminepentaacetic acid (DTPA), leveraging intermolecular hydrogen bonding. Hydrogen bonding crosslinking endowed the hydrogel with outstanding properties, including mechanical strength far superior to traditional hydrogels (fracture stress: 515 ± 9 kPa, fracture strain: 1374% ± 20%), good adhesion to various substrates (especially biological tissues), and a higher swelling ratio (1519%).

The hydrogen bonding crosslinking system exhibits both significant advantages and inherent limitations. In terms of advantages, the dynamic reversibility of hydrogen bonds endows hydrogels with self-healing capability and injectability, and the hydrogen bonding network can serve as sacrificial bonds to efficiently dissipate energy, allowing the material to achieve higher mechanical strength while maintaining high stretchability. The abundant polar groups within the system facilitate enhanced interfacial adhesion between the material and biological tissues, while also conferring good biocompatibility. Furthermore, hydrogen bonds can synergistically modulate swelling behavior and drug release kinetics, endowing the material with environmental responsiveness. However, as relatively weak non-covalent interactions, hydrogen bonds alone are insufficient to construct a stable network with adequate mechanical strength. More critically, competitive binding by water molecules and elevated temperatures can lead to hydrogen bond dissociation, resulting in a significant degradation of the mechanical properties of the hydrogel under conditions of high water content or high temperature. This inherent trade-off between strength and dynamics represents a core challenge that needs to be addressed in the design of hydrogen-bonded hydrogels.

#### 2.1.3. Hydrophobic Interaction

**Figure 3:**
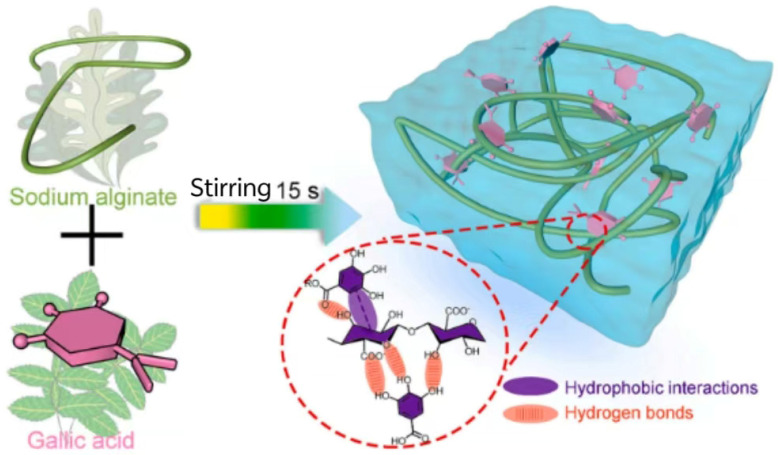
Schematic diagram of the preparation process of SA-GA hydrogels [12]. Copyright 2023, Elsevier.

Hydrophobic interaction refers to the mutual attraction and aggregation between non-polar molecules or polymer segments, and represents an important crosslinking method for preparing sodium alginate-based composite hydrogels. It is mainly divided into two types: direct hydrophobic aggregation and hydrophobic interactions mediated by grafted hydrophobic groups. Chen et al. [12] successfully prepared a Polyphenol–Sodium Alginate Injectable Hydrogel (PBSIH) by mixing the polyphenol gallic acid (GA) with the biopolymer sodium alginate (SA) utilizing hydrophobic interactions, as shown in Figure 3. Hydrophobic interactions formed between the benzene rings of GA and the pyranose rings of SA, promoting rapid hydrogel formation. This preparation method based on hydrophobic interactions enabled the hydrogel to form within 15 s, and endowed it with excellent self-healing properties, adhesiveness, and rapid and complete drug release capability. Jing et al. [13] successfully synthesized alginate-based semi-interpenetrating network (NaAlg/PAM semi-IPN) and double-network (CaAlg/PAM DN) hydrogels via hydrophobic interactions. Through micellar copolymerization of acrylamide and methyl stearate, hydrophobic micelles served as crosslinking points to construct the hydrogel network. The introduction of hydrophobic interactions endowed the hydrogels with outstanding mechanical strength, toughness, rapid self-recovery ability, and good fatigue resistance. Ribeiro et al. [14] designed a multifunctional sandwich-like system for treating wound infections, consisting of an outer layer of polycaprolactone (PCL) fibrous membrane, a middle layer of sodium alginate (SA) hydrogel loaded with ampicillin (Amp), and an inner layer of PCL and polyethylene glycol (PEG) fibrous membrane. The hydrophobic nature of PCL serves as a key characteristic of the outer fibrous membrane, aiming to prevent external liquid contaminants from entering the wound. The hydrophobicity of the PCL outer layer synergizes with the hydrophilicity of the PCL/PEG inner layer, enabling the entire system to effectively manage wound moisture, provide external protection, and promote targeted drug release. Conzatti et al. [15] performed hydrophobic modification on the surface of highly hydrophilic alginate/chitosan polyelectrolyte complexes (PECs). After modification, the swelling capacity of the material decreased but still maintained a water absorption rate of approximately 1000%. The hydrophobic layer can act as an effective barrier, significantly enhancing the material’s resistance to penetration by enzymatic media and reducing fluid leakage. Among these, the PCL coating exhibited poorer stability in an enzymatic environment, while the PLA coating demonstrated superior structural stability and anti-leakage performance.

Hydrophobic interactions offer multiple advantages in the synthesis of hydrogels, primarily serving as an efficient driving force for physical crosslinking that rapidly induces molecular chain self-assembly to construct three-dimensional networks, thereby endowing hydrogels with excellent self-healing properties and dynamic responsiveness without the need for introducing chemical crosslinking agents. The dynamic reversible structure of hydrophobic domains can also act as energy dissipation sites, effectively enhancing the mechanical toughness and fatigue resistance of the material. Furthermore, hydrophobic interfaces can establish robust protective barriers, which can both block the intrusion of external contaminants and achieve targeted and controlled drug release. However, hydrophobic interactions also have some potential limitations. Hydrophobic modification typically reduces the hydrophilic and water-absorbing characteristics of the material, leading to decreased swelling performance, making it difficult to meet the high fluid absorption requirements of wound dressings. Certain hydrophobic components, such as PCL, are prone to degradation in physiological enzymatic environments, making it challenging to maintain the long-term stability of the hydrophobic barrier. Moreover, excessive hydrophobicity of the material can induce protein adsorption and conformational changes, interfering with interfacial biocompatibility and even triggering non-specific immune responses.

#### 2.1.4. Electrostatic Interaction

**Figure 4:**
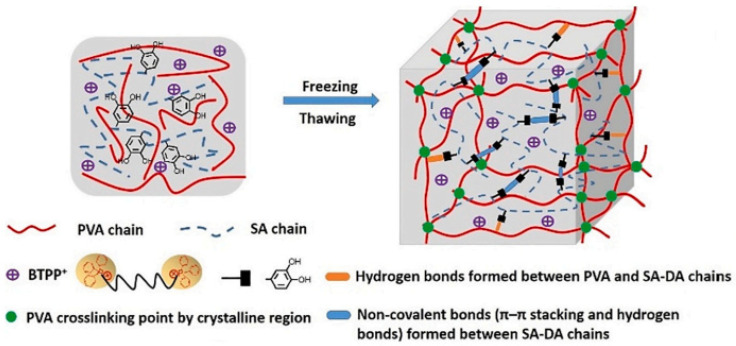
Schematic diagram of the preparation of PVA/SA-DA/BTPP^+^ hydrogels via electrostatic bonding [18]. Copyright 2021, Elsevier.

Sodium alginate is a natural anionic polyelectrolyte, and the carboxyl groups (-COO^−^) on its molecular chains confer a high density of negative charge in aqueous solution. When mixed with positively charged cationic polyelectrolytes (such as chitosan, gelatin, polylysine, etc.), the two self-assemble through electrostatic attraction to form polyelectrolyte complexes, where oppositely charged chain segments gradually associate and crosslink, constructing a stable three-dimensional hydrogel network [16]. Wu et al. [17] used sodium methacrylate alginate (MASA) and carboxymethyl chitosan (CMCS) as raw materials to prepare a double-network (DN) hydrogel by exploiting the electrostatic interaction between the carboxyl groups of MASA and the amino groups of CMCS under an acetic acid atmosphere. Electrostatic interactions significantly enhanced the mechanical properties of the hydrogel, increased its light transmittance, endowed the hydrogel with excellent water absorption and pH-sensitive drug release behavior, and improved the structural uniformity of the hydrogel by partially replacing hydrogen bonds. Lei et al. [18] stably immobilized a butyltriphenylphosphonium cation (BTPP^+^) bactericide onto a polyvinyl alcohol (PVA)/sodium alginate–dopamine (SA-DA) hydrogel via electrostatic interactions, as shown in Figure 4. This endowed the hydrogel with long-lasting antibacterial activity, significantly improved the hemostatic ability of the hydrogel, and enhanced its compressive stress due to the increased crosslinking density. Zhuo et al. [19] combined self-assembled chitosan-sodium alginate nanofibers (CSNFs) with tannic acid (TA) and polyvinyl alcohol (PVA) to prepare ultra-strong nanofiber-reinforced composite hydrogels. The formation of CSNFs relied on the strong electrostatic interaction between chitosan and sodium alginate, while TA formed electrostatic interactions with the -NH_3_^+^ groups of CSNFs by releasing phenolate anions. This significantly enhanced the overall mechanical properties of the composite hydrogel, thereby improving its strength and toughness. Jiang et al. [23] prepared cationic nanocellulose (CCNF) from *Astragalus* residue and mixed it with anionic sodium alginate (SA) to fabricate CCNF-SA hydrogel dressings via electrostatic interactions. The electrostatic interactions imparted moderate viscosity to the dressing, facilitating wound coverage. Furthermore, the electrostatic interactions promoted the formation of a nanofibrous porous structure in the dressing, significantly improving oxygen permeability, which is beneficial for wound healing.

As a physical crosslinking approach, electrostatic interactions exhibit multiple advantages in hydrogel systems: they can drive the self-assembly of components and construct ordered network structures under mild conditions, effectively enhancing mechanical strength and toughness; their reversibility and dynamics endow materials with intelligent functions such as pH responsiveness and controlled drug release; meanwhile, electrostatic adsorption can stably anchor functional molecules (e.g., bactericides) within the gel matrix, achieving sustained biological activity, and can synergize with other non-covalent bonds such as hydrogen bonds and hydrophobic interactions to produce enhanced effects. However, electrostatic interactions also have inherent limitations: their strength is highly dependent on environmental conditions such as pH and ionic strength, and they are easily shielded or disrupted under high-salt or extreme pH conditions, leading to destabilization of the network structure; moreover, the mixing of positively and negatively charged components is prone to local over-complexation or uneven aggregation, imposing high demands on precise control of the preparation process; furthermore, relying solely on electrostatic crosslinking often fails to achieve sufficient long-term mechanical stability, and it is usually necessary to combine it with covalent crosslinking or other physical crosslinking methods to meet the demands of practical applications.

### 2.2. Chemical Cross-Linking

Chemical crosslinking refers to the formation of a three-dimensional network structure by connecting monomers or polymers to each other through covalent bonds. By modifying the functional groups of sodium alginate, sodium alginate-based polymers are prepared, which are then linked via chemical bonds to form sodium alginate-based composite hydrogels. Chemical crosslinking typically requires the addition of crosslinking agents, commonly including glutaraldehyde, epichlorohydrin, and the like. Residues of such chemical crosslinking agents may remain after use, potentially causing environmental impact. Compared with physical crosslinking, chemical crosslinking offers greater stability [24]. Overall, chemical crosslinking endows sodium alginate-based composite hydrogels with superior structural stability, mechanical strength and functional ductility via forming stable covalent bonds. Methods such as Schiff base crosslinking, photo-crosslinking, radiation crosslinking and enzymatic crosslinking enable rapid shaping, precise regulation of physicochemical properties and efficient incorporation of bioactive substances, and exhibit outstanding performances in antibacterial, anti-inflammatory and tissue regeneration-promoting effects. Nevertheless, chemical crosslinking still faces several challenges, including residual crosslinkers, unsatisfactory biosafety, difficult regulation of crosslinking density and high preparation cost. Additionally, enzymatic crosslinking suffers from environment-sensitive enzyme activity and slow gelation rate. Accordingly, to facilitate its clinical translation, it is essential to strictly evaluate the biosafety of chemical crosslinkers, optimize preparation processes to reduce toxic residues, and guarantee the long-term stability and functionality of materials in vivo, so as to realize their extensive applications in wound repair. The advantages and disadvantages of various chemical crosslinking methods are shown in Table 2.

#### 2.2.1. Schiff Base Crosslinking

**Figure 5:**
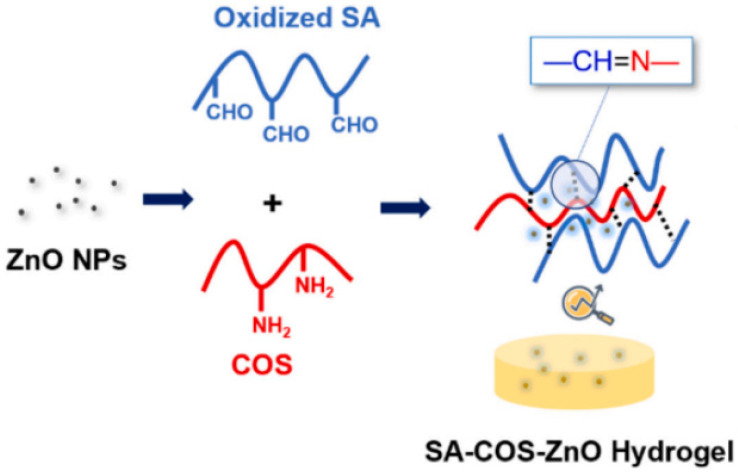
Schematic illustration of SA-COS-ZnO hydrogel fabrication [41]. Copyright 2021, Elsevier.

Schiff base crosslinking refers to the reaction between aldehyde-containing oxidized sodium alginate and amino-containing polymers or small-molecule crosslinkers under mild conditions to form dynamic reversible imine bonds through the condensation of amino and aldehyde groups, thereby constructing a three-dimensional crosslinking network. This crosslinking method exhibits good biocompatibility, pH responsiveness, self-healing properties, and moderate thermal stability [25]. Yu et al. [26] added NaCl to weaken electrostatic interactions, ensuring that *N-*(2-hydroxypropyl)-3-trimethylammonium chitosan chloride (HACC) and oxidized sodium alginate (OSA) uniformly synthesized hydrogels via Schiff base reactions. The resulting hydrogel possessed a uniform porous structure, enhanced mechanical elasticity, good swellability, biodegradability, water retention, broad-spectrum antibacterial activity, excellent cytocompatibility, and drug sustained-release capability. Wang et al. [27] fabricated injectable and self-healing hydrogels by mixing oxidized sodium alginate (OSA) and quaternized chitosan (QCS) at room temperature followed by pH adjustment. The formed Schiff base bonds optimize the network structure of hydrogels and improve their thermal stability and viscoelasticity, which reduces the swelling rate, achieves controlled release of curcumin, and endows the hydrogels with excellent injectability and self-healing performance. Yang et al. [28] prepared an injectable verteporfin-carboxymethyl chitosan-oxidized sodium alginate hydrogel (VP-CMCS-OSA), using verteporfin (VP)-conjugated carboxymethyl chitosan (CMCS) and oxidized sodium alginate (OSA) as raw materials. The Schiff base linkage between VP-CMCS and OSA was key to hydrogel formation. This hydrogel exhibited outstanding self-healing ability, high tissue adhesion, good stretchability, biocompatibility, and enabled long-term sustained release of verteporfin. Zhang et al. [41] prepared verteporfin-carboxymethyl chitosan oligosaccharide sodium alginate (SA-COS-ZnO) composite hydrogels using oxidized sodium alginate (SA), chitosan oligosaccharide (COS), and zinc oxide nanoparticles (ZnO NPs) as raw materials through a spontaneous Schiff base reaction between the aldehyde groups of SA and the amino groups of COS, as shown in Figure 5. The Schiff base crosslinking endowed the hydrogel with a porous three-dimensional network structure, imparting good water retention capacity, appropriate porosity and swelling degree, as well as ideal water vapor transmission rate.

Schiff base crosslinking demonstrates significant advantages in hydrogel preparation, primarily reflected in its ability to rapidly form stable and reversible crosslinking networks, thereby endowing hydrogels with properties such as self-healing, injectability, controllable gelation time, enhanced mechanical strength, and elasticity. Furthermore, the dense network structure formed by Schiff base bonds facilitates sustained and controlled drug release, and enables the regulation of the hydrogel’s microstructure and swelling behavior by adjusting the component ratios. However, Schiff base crosslinking also has some potential limitations. For example, strong electrostatic interactions in certain systems may lead to flocculation, which needs to be addressed by adding shielding agents. In addition, the Schiff base reaction may consume active sites, thereby affecting some inherent properties of the resulting hydrogel. Therefore, future research should focus on developing novel non-toxic or low-toxic aldehyde/amino precursors and optimizing reaction conditions to achieve more efficient and stable Schiff base crosslinking, while minimizing side effects and negative impacts on material properties.

#### 2.2.2. Photocrosslinking

**Figure 6:**
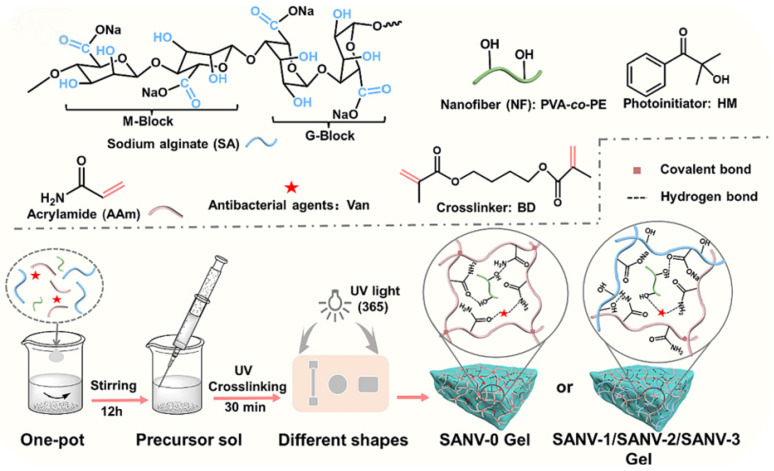
Schematic illustration of hydrogel fabrication [42]. Copyright 2024, Elsevier.

Photocrosslinking refers to a technique that introduces photosensitive groups into the molecular chains of sodium alginate through chemical modification, or utilizes a photosensitive auxiliary system, to rapidly trigger crosslinking reactions upon irradiation with light of a specific wavelength, thereby connecting the sodium alginate molecular chains to form a three-dimensional network structured hydrogel [29]. This technique offers advantages such as rapid fabrication, mild conditions, and precise structural tunability, and has become an important method for preparing sodium alginate-based composite hydrogels. Vieira et al. [30] conducted an in-depth study on the effect of photosensitized crosslinking on the properties of methacrylated alginate (AlgMA) hydrogels, particularly when poly(limonene) (PLM) was incorporated. By precisely controlling ultraviolet (UV) light irradiation, they achieved the formation of the gel network and found that the PLM content directly regulated the gel’s hardness and crosslinking efficiency. Building on the aforementioned fundamental research, Vieira et al. [31] applied photocrosslinking technology to the practical application development of methacrylated alginate/poly(limonene) (AlgMA/PLM) hydrogels. They formed a stable three-dimensional network of AlgMA via UV light irradiation and successfully integrated PLM into it. This endowed the hydrogel with excellent mechanical strength, a porous structure, and good biocompatibility, achieving effective release of PLM. In vivo experiments significantly promoted wound healing and tissue regeneration, validating its potential as an efficient wound dressing. Cao et al. [32] utilized photocrosslinking technology to prepare a gelatin methacryloyl (GelGMA)-oxidized sodium alginate (OSA) double-network hydrogel loaded with gentamicin sulfate (GS), as shown in Figure 6. Photocrosslinking served as a secondary reinforcement method. After the initial network formation between GelGMA and OSA, irradiation with a 365 nm UV lamp for 1 min initiated the polymerization of methacryloyl groups on the GelGMA molecular chains. This process significantly enhanced the mechanical strength of the hydrogel and endowed it with good elasticity, swelling performance, and drug sustained-release capability, effectively inhibiting infection in diabetic wounds and promoting granulation tissue growth. Zhang et al. [42] prepared a sodium alginate (SA)-based multifunctional composite hydrogel using UV photopolymerization technology. This technique initiated the formation of a covalent crosslinking skeleton of acrylamide via UV light, effectively enhancing the hydrogel’s excellent stretchability, compressibility, and tissue adhesion, while also demonstrating strong antibacterial and procoagulant functions, making it an ideal wound dressing material.

The advantages of photocrosslinking lie in its ability to precisely adjust the physicochemical properties of the gel, such as hardness, crosslinking efficiency, and pore structure, thereby endowing hydrogels with excellent mechanical strength, elasticity, and multifunctionality. It can effectively integrate bioactive substances, achieve controlled release of drugs or antioxidants, and promote tissue regeneration and wound healing. However, its potential drawbacks are also evident. For example, excessive concentrations of photoinitiators may affect cytocompatibility, and the introduction of certain additives (such as PLM) may reduce crosslinking efficiency, consequently impacting the final performance of the gel.

#### 2.2.3. Radiation Crosslinking

Radiation crosslinking refers to a green, additive-free, and crosslinker-free crosslinking technology in which polymer molecular chains generate free radicals under the action of ionizing radiation (γ-rays, electron beams, X-rays) and undergo covalent crosslinking, thereby forming a stable three-dimensional network structure [33]. Nurlidar et al. [34] successfully developed a ciprofloxacin-encapsulated polyethylene glycol diacrylate/alginate (PEGDA/ALG) composite hydrogel using gamma irradiation technology. Radiation induces the radiolysis of water molecules to produce highly reactive free radicals, prompting the formation of macromolecular free radicals from polymer chains, which then combine with each other to construct a stable three-dimensional covalent network. This approach simultaneously achieved gel synthesis and drug loading. Song et al. [35] prepared debridement hydrogels using gelatin, sodium alginate, and sodium carboxymethyl cellulose as matrices via γ-radiation crosslinking. The study showed that the physicochemical properties of the hydrogels were regulated by the weight ratios of the raw materials, radiation dose, and dose rate. Under optimal conditions, the swelling ratio of the hydrogels could reach 30 times their own dry weight, and they exhibited good biosafety. Ren et al. [36] prepared marine polysaccharide nanocomposite hydrogels loaded with quercetin and silver nanoparticles using an electron beam irradiation strategy. Radiation crosslinking replaced chemical crosslinking agents, utilizing reactive intermediates generated by high-energy electron excitation to rapidly tighten the polysaccharide molecular chains and induce chemical bonding, thereby encapsulating the active components. The radiation-induced dense network structure ensured rapid bactericidal release of silver ions and long-term antioxidant sustained release of quercetin, enhancing the hydrogel’s biocompatibility and biofilm clearance capability. Mignon et al. [43] synthesized high-strength transparent hybrid hydrogels using electron beam initiation technology. Radiation crosslinking, employing high-energy electrons generated by a linear accelerator, directly initiated polymerization of monomers without the need for toxic photoinitiators, achieving uniform crosslinking of the polymer network. The resulting hydrogels exhibited extremely high mechanical storage moduli and excellent light transmittance, while maintaining high gel fractions and strong water-swelling properties, along with the clinically required sterile characteristics.

Radiation crosslinking technology exhibits multiple advantages in the preparation of sodium alginate-based composite hydrogels. It avoids the use of toxic chemical initiators and enables the simultaneous accomplishment of gel fabrication, drug encapsulation, or sterilization, thereby simplifying the process and improving efficiency. By precisely controlling the radiation parameters, the physicochemical properties of the hydrogels, such as swelling ratio, mechanical strength, and pore structure, can be effectively regulated, thereby influencing drug release kinetics and bioactivity. Furthermore, radiation crosslinking endows the hydrogels with good biocompatibility, antibacterial properties, antioxidant activity, and high transparency, offering broad application potential in fields such as drug delivery, wound healing, and biomedical imaging. However, precise control of radiation dose and dose rate is critical to material performance; excessive or improper radiation may lead to polymer degradation or suboptimal properties. Therefore, future research should further understand the effects of radiation on polymer structures and bioactive substances, optimize radiation protocols, ensure material performance while minimizing potential biological risks, and promote its widespread applications in drug delivery, wound healing, and biomedical imaging.

#### 2.2.4. Enzymatic Crosslinking

Enzymatic crosslinking refers to the process of constructing a three-dimensional network hydrogel using natural polysaccharide sodium alginate (SA) as a matrix, with the aid of catalysis by specific enzymes or through enzyme-catalyzed reactions that facilitate the formation of covalent bonds between sodium alginate and other polymers [37]. Cadamuro et al. [38] fabricated alginate-gelatin self-healing hydrogels to simulate the extracellular matrix of soft tissues. With the synergistic catalysis of horseradish peroxidase (HRP) and hydrogen peroxide (H_2_O_2_), tyrosine residues in gelatin were enzymatically coupled with tyramine residues introduced into the alginate skeleton, thereby constructing stable covalent cross-linking networks between the two molecules. Wang et al. [39] successfully prepared hydrogels using the horseradish peroxidase/hydrogen peroxide (HRP/H_2_O_2_) enzymatic system. Enzymatic crosslinking significantly increased the viscosity, storage modulus, and loss modulus of the hydrogel by at least a hundredfold, and exhibited excellent thixotropic recovery ability as well as remarkable strength and toughness. Kolour et al. [4] utilized microbial transglutaminase (mTG) as an enzymatic crosslinker to prepare biodegradable gelatin/alginate semi-interpenetrating polymer network (semi-IPN) hydrogel wound dressings loaded with curcumin. mTG enzymatic crosslinking forms covalent bonds between gelatin molecules by catalyzing an acyl transfer reaction between the γ-carboxamide group of glutamine residues and the ε-amino group of lysine residues, forming ε-(γ-glutamyl)-lysine isopeptide bonds. Feng et al. [44] developed a novel catechol-conjugated alginate (C-Alg) hydrogel system. Laccase catalyzed the chemical crosslinking of catechol groups, thereby providing progressive stability. Laccase, as a multicopper oxidase, utilizes non-toxic oxygen as an oxidant to catalyze the oxidation of catechol groups, forming a stronger covalent crosslinking network.

The advantages of enzymatic crosslinking lie in its ability to endow hydrogels with excellent structural stability and enhanced mechanical properties (such as viscosity, storage modulus, compressive modulus, strength, toughness, elasticity, and stiffness), as well as to effectively regulate the material’s self-healing capability, injectability, gelation time, cytocompatibility, biodegradability, water absorption/dehydration performance, and drug release behavior. Furthermore, enzymatic crosslinking can promote cell migration and proliferation, and improve the adhesion of the material to tissues. However, enzymatic crosslinking also has some limitations. For example, certain enzymes (such as HRP) may require toxic H_2_O_2_ as a cofactor, which limits their application under physiological conditions. Additionally, the enzyme concentration and reaction conditions need to be precisely optimized to avoid adverse effects on the polymer matrix or to prevent compromised crosslinking efficiency.

## 3. Drug Release Mechanism of SA-Based Composite Hydrogels

The drug release behavior of hydrogels is critical to their therapeutic efficacy. This chapter provides an in-depth discussion of the drug release mechanisms of sodium alginate-based composite hydrogels. The release of sodium alginate-based composite hydrogels refers to the process by which, after forming a three-dimensional network structure through physical or chemical crosslinking of sodium alginate with other polymers, the loaded active substances such as drugs, food functional components, or agricultural nutrients are delivered at a predetermined rate in the target environment via mechanisms including physical diffusion, swelling, degradation, or stimulus responsiveness [45]. Based on the behavioral characteristics and regulatory modes of drug release, the release mechanisms of sodium alginate-based composite hydrogels can be classified into three types: sustained release, controlled release, and stimuli-triggered release. Among these, sustained release focuses on prolonging the release period and reducing burst release; controlled release emphasizes precise regulation of release rate and targeted delivery; stimuli-triggered release achieves on-demand release through external stimuli (pH, temperature, light, etc.). The three categories are not mutually exclusive, as many hydrogel systems exhibit multiple release characteristics; for example, pH-responsive hydrogels also possess controlled release capability. The drug release behavior of sodium alginate-based composite hydrogels can be classified into sustained release, controlled release, and stimuli-responsive release, with significant differences among the three in terms of regulatory modes and application scenarios. The characteristics, advantages, and limitations of the three release mechanisms are shown in Table 3.

### 3.1. Sustained Release

**Figure 7:**
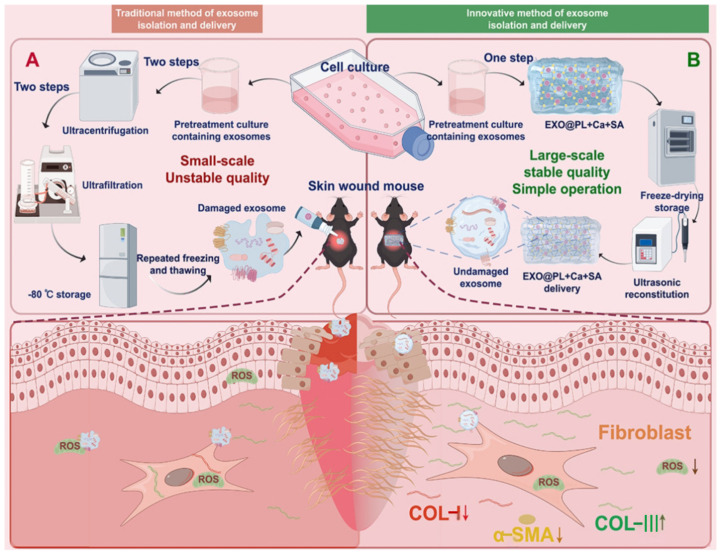
Schematic illustration of isolation-to-delivery hydrogel for exosome loading and enhanced wound healing: (**A**) Traditional method for exosome isolation and delivery, (**B**) Innovative approach for exosome isolation and delivery [47]. Copyright 2026, Elsevier.

The sustained drug release of sodium alginate-based composite hydrogels primarily relies on three synergistic mechanisms: network diffusion, swelling-driven release, and degradation-mediated dissociation. By regulating key parameters such as sodium alginate concentration, crosslinking density, and the type of composite components, the pore size and structural compactness of the gel network can be effectively modified. Through the synergistic action of physical diffusion, gel swelling, and biodegradation, a slow, continuous, and stable release of active substances is achieved, ultimately reducing initial burst release and prolonging the release period to meet the clinical application requirements of long-term delivery [66]. Gatabi et al. [46] developed a metformin/bentonite nanocomposite hydrogel based on sodium alginate. The drug release behavior of this hydrogel followed the Korsmeyer–Peppas model, with the release process obeying typical Fickian diffusion law. The drug release rate was primarily governed by the permeation and diffusion of drug molecules within the hydrogel matrix, achieving a smooth and long-lasting sustained release effect. Yang et al. [47] designed a multifunctional bilayer polysaccharide hydrogel aimed at efficiently separating and continuously releasing exosomes. This hydrogel achieved exosome separation and controlled release through freeze-drying combined with ultrasound-assisted dissolution technology. The release behavior of exosomes conformed to the Higuchi model, enabling continuous release for at least 7 days. Chen et al. [48] prepared slow-setting graphene oxide (GO)/alginate hydrogels loaded with platelet-rich plasma (PRP). Glucono-δ-lactone was used to slowly release calcium ions for gradual gelation, forming a three-dimensional porous structure with uniform pore size and high water content. This structure not only effectively loaded PRP and maintained platelet activity and its growth factors but also achieved sustained release of platelet-derived growth factor (PDGF) through slow in vitro degradation characteristics. Huang et al. [49] prepared a bioactive multi-layer core–shell fiber hydrogel loaded with platelet-rich plasma (PRP), as shown in Figure 7. The unique multi-layer core–shell structure of this hydrogel provided steric hindrance, effectively prolonging the release time of growth factors (GFs). The release behavior of GFs from the three-layer core–shell fiber hydrogel (3LSCF-EP gel) followed zero-order release model, while that from the two-layer core–shell fiber hydrogel (2LSCF-EP gel) primarily adhered to Fickian diffusion. This design enabled long-term sustained release of growth factors (GFs), thereby reducing the dosing frequency by 33% during treatment.

The significant advantages and potential limitations of sustained release strategies in biomedical applications. Sustained release technology effectively prolongs the duration of action of active substances (such as metformin, exosomes, and growth factors) through sophisticated material design and regulation of release mechanisms, thereby reducing dosing frequency and improving therapeutic outcomes, including accelerated wound healing, promoted tissue regeneration, and reduced inflammatory responses. However, the preparation process of sustained-release systems remains relatively complex. Rational regulation of hydrogel structure and crosslinking parameters is required to avoid initial burst release or insufficient late-stage release. Meanwhile, the biocompatibility and degradability of the materials themselves must be strictly evaluated to ensure long-term application safety.

### 3.2. Controlled Release

**Figure 8:**
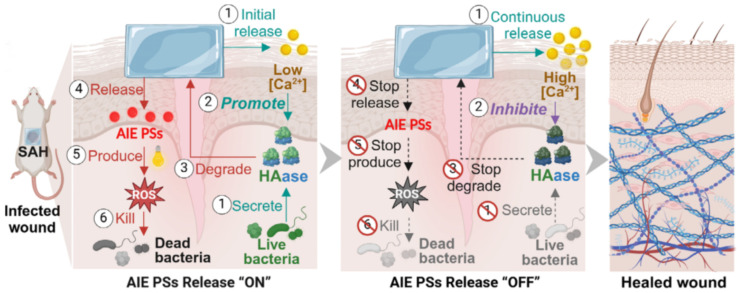
Controlled release of ROS for infected wound healing by Ca^2+^-responsive and light-activated SAH dressing [50].

The controlled release mechanism of sodium alginate-based composite hydrogels involves precisely regulating the drug diffusion rate within the gel by adjusting the crosslinking density, porous structure, and matrix component content of the gel, or by employing polymer grafting modification to enhance gel stability, slow erosion, and respond to environmental pH signals to alter the network compactness, ultimately achieving long-term and controllable sustained drug release. Cui et al. [50] developed a switchable antibacterial hydrogel (SAH) loaded with an aggregation-induced emission (AIE) photosensitizer triphenylamine–thiophene–pyridinium derivative (TTPy-NH_2_), as shown in Figure 8. The core of its controlled release is calcium ion-mediated bidirectional regulation of bacterial hyaluronidase (HAase) activity. Low concentrations of calcium ions stabilize the enzyme conformation and enhance its activity, triggering hyaluronic acid degradation and subsequent photosensitizer release, whereas high concentrations of calcium ions induce enzyme salting-out and inactivation, achieving automatic termination of release. Liu et al. [51] constructed a polyelectrolyte complex hydrogel based on sodium alginate-graft-poly(*N*-isopropylacrylamide) (Alg-g-PNIPAAm) and chitosan, which enables long-term and smart controlled release of rhodamine B. Under neutral conditions, the strong electrostatic attraction between Alg-g-PNIPAAm and chitosan restricted drug diffusion; when the temperature exceeded the lower critical solution temperature (LCST) of PNIPAAm, the PNIPAAm segments underwent hydrophobic collapse, enhancing the stability of the gel network and reducing pore size. Ma et al. [52] developed a pH-responsive double-network biopolysaccharide hydrogel (DP-CS-SA) with enhanced self-healing capability. The controlled release mechanism of this gel relied on the dynamic balance between two effects. Under acidic conditions, the Schiff base bonds in the gel undergo hydrolytic cleavage, while the electrostatic interactions within the system are significantly enhanced; the coordination between these two effects achieves stable controlled release. This design not only endowed the gel with excellent injectability and self-healing properties but also successfully achieved sustained release of baicalin, demonstrating outstanding antibacterial and biocompatibility effects. Jebessa et al. [53] prepared a sodium alginate-polyvinylpyrrolidone (SA/PvP) hydrogel loaded with tinidazole (TNZ) via ionotropic gelation. This hydrogel achieved controlled drug release through its swelling behavior and diffusion-controlled mechanism, maintaining low release in simulated gastric environments while effectively releasing the drug in simulated colonic environments, thereby achieving colon-targeted release and exhibiting significant antibacterial activity.

Controlled-release hydrogel systems exhibit significant advantages. They enable precise on-demand drug release in response to specific biological signals (such as enzyme activity, pH) or external stimuli (such as temperature, near-infrared light), thereby maximizing therapeutic efficacy while minimizing side effects on healthy tissues. For example, they can avoid excessive reactive oxygen species damage through bidirectional regulation mechanisms, or utilize photothermal effects to achieve accelerated drug release and enhance antibacterial activity. However, these complex controlled-release mechanisms may also present challenges, including the complexity of the preparation process, the difficulty in precisely balancing different stimulus responses, and the potential for off-target effects or stability issues in complex biological environments. Furthermore, certain mechanisms may depend on specific environmental conditions, limiting their universality.

### 3.3. Stimuli-Triggered Release

Stimuli-triggered release refers to the on-demand and controllable release of loaded drugs from hydrogels upon exposure to specific external stimulus signals (such as pH, temperature, light, etc.), achieved through swelling, shrinkage, degradation, or the breakage and recombination of crosslinking bonds within the hydrogel network structure. Depending on the type of triggering signal, it can be classified into three main categories: light-responsive release, pH-responsive release, and temperature-responsive release. Compared with sustained release and controlled release, stimuli-triggered release can more precisely match the dynamic changes of the complex wound microenvironment (such as the low pH of infected wounds, excessive heat at inflamed sites, etc.), thereby meeting the therapeutic requirement of ‘on-demand delivery’ [67]. However, each type of stimuli-responsive mechanism has its own limitations. Although light-responsive release offers precise controllability, it suffers from limited light penetration depth, requires additional equipment, and carries the risk of phototoxicity. pH-responsive release is prone to instability caused by in vivo pH fluctuations, and the long-term biosafety of certain responsive components remains to be evaluated. Temperature-responsive release faces challenges such as a narrow phase transition range, unstable release behavior, and potential cytotoxicity caused by burst release. Therefore, future research should focus on developing intelligent hydrogels with multi-signal synergistic responses to overcome the limitations of single-stimulus responsiveness, enabling more precise and safer drug delivery, and exploring the integration of biosensing with drug release to achieve real-time feedback and intelligent regulation for wound repair.

#### 3.3.1. Light-Responsive Release

**Figure 9:**
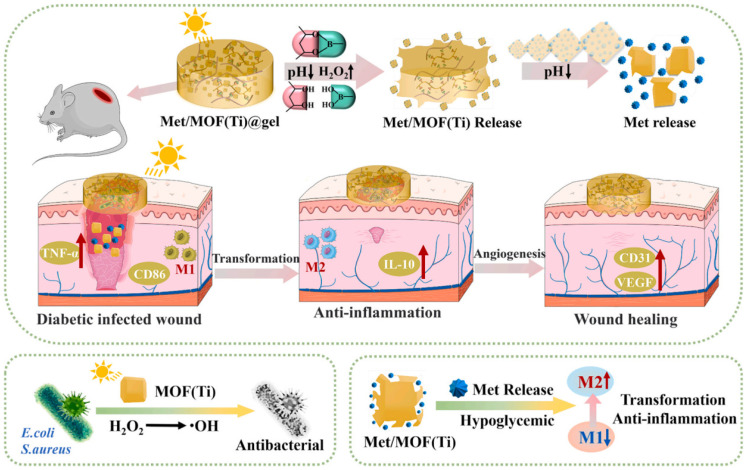
Schematic of drug release behavior and chronic diabetic wound healing mechanism of Met/MOF(Ti)@gel dressing [55]. Copyright 2025, Elsevier.

Light-responsive release of sodium alginate-based composite hydrogels utilizes natural polysaccharide sodium alginate as the core matrix to construct a three-dimensional network hydrogel carrier. By incorporating light-responsive components or introducing photosensitive modifications, active substances such as drugs are encapsulated within the gel network. The mechanisms of light-responsive release vary significantly depending on the triggering light source. Ultraviolet (UV) light typically induces isomerization or chemical bond cleavage of photosensitive groups (e.g., azobenzene, o-nitrobenzyl), directly altering the gel network structure or disrupting crosslinking points to achieve controlled drug release. Near-infrared (NIR) light primarily relies on light-energy conversion media such as upconversion nanoparticles to convert NIR into UV or visible light, which then activates photosensitizers to generate reactive oxygen species (ROS) or induce photothermal effects, indirectly modulating the degradation rate or network structure of the hydrogel. Compared with UV light, NIR light offers advantages of greater tissue penetration depth and lower phototoxicity, making it more suitable for applications such as deep wound healing in vivo [68]. Yang et al. [54] developed a gold nanorod double-crosslinked hydrogel (D-hydrogel@AuNR) that achieved rapid response to NIR light by embedding gold nanorods (AuNRs) within the polymer network. Upon irradiation with 808 nm NIR, the AuNRs rapidly underwent photothermal conversion, and the generated heat induced microphase separation of the polymers inside the hydrogel, leading to volume shrinkage and efficient squeezing out of the encapsulated gentamicin sulfate (GS). This process achieved up to 75% GS release within 60 s. Tian et al. [55] designed a metformin/titanium-based metal–organic framework hydrogel composite dressing (Met/MOF(Ti)@gel) that ingeniously combined the visible-light-activated photocatalytic properties of MOF(Ti) with the dual sensitivity of the hydrogel to acidic and H_2_O_2_ conditions. Under visible light irradiation, MOF(Ti) catalyzed the generation of potent antibacterial •OH radicals from H_2_O_2_; meanwhile, the acidic and H_2_O_2_ conditions of the diabetic wound microenvironment promoted rapid disintegration of the hydrogel, accelerating the release of metformin (Met) and MOF(Ti). Ma et al. [56] developed a novel light-responsive injectable hydrogel dressing (CSMA/SI/AuNP hydrogel). Light irradiation induced a liquid-to-solid gel transition, establishing a stable “drug reservoir” at the wound site, followed by sustained release of soy isoflavones and gold nanoparticles through diffusion and biodegradation of the gel. Zhang et al. [57] developed a functionalized silica nanoparticle@deferoxamine-hyaluronic acid chitosan hydrogel@dexamethasone composite nanogel (FSNs@DFO-H@Dex), as shown in Figure 9. The drug release was regulated by NIR light: the functionalized silica nanoparticles (FSNs) generated a photothermal effect upon NIR irradiation, raising the local temperature and thereby accelerating the diffusion and release of encapsulated deferoxamine (DFO), while dexamethasone (Dex) within the hydrogel was also continuously released.

Light-responsive release technology exhibits significant advantages, primarily reflected in its precise controllability and rapid responsiveness. Through external light irradiation, on-demand drug release at specific times and locations can be achieved, effectively avoiding the systemic side effects associated with traditional drug administration methods, while allowing flexible adjustment of the release rate according to therapeutic needs. Furthermore, the introduction of photothermal effects can synergistically enhance therapeutic outcomes, such as promoting wound healing or exerting antibacterial effects. However, this technology also has limitations, such as the limited depth of light penetration, which may hinder the treatment of deep tissues, and the requirement for additional light irradiation equipment, which increases operational complexity.

#### 3.3.2. pH-Responsive Release

The core mechanism of pH-responsive hydrogel release involves modulating the ionization state of functional groups within the hydrogel through changes in the external environmental pH, thereby altering the compactness or swelling degree of the gel network and ultimately achieving controlled release of encapsulated drugs [69]. He et al. [58] designed a pH-responsive sodium alginate hydrogel microsphere for targeted delivery of Lactobacillus reuteri (L.r) to treat ulcerative colitis in mice. This hydrogel ingeniously utilized the properties of zeolitic imidazolate framework-67 (ZIF-67). It decomposes and releases cobalt ions in the gastric acid environment, which can perform in situ crosslinking with sodium alginate to form a dense gel barrier and effectively protect probiotics from gastric acid damage. After entering the neutral intestinal environment, the weakened coordination bonds cause the gel to swell and disintegrate, achieving precise release of probiotics. Wu et al. [59] developed a pH-sensitive dual-release hydrogel based on oxidized sodium alginate (OSA) and carboxymethyl chitosan (CMCS), specifically designed for diabetic wound healing. The release mechanism of this hydrogel cleverly utilizes the Schiff base bonds within it, which undergo accelerated hydrolysis under acidic conditions. In the acidic microenvironment of diabetic wounds, the hydrogel rapidly releases diclofenac sodium (DS) with antibacterial and anti-inflammatory effects, while simultaneously and slowly releasing astragaloside liposomes (AL) with antioxidant and pro-angiogenic effects under sustained acidic conditions. Li et al. [60] prepared a pH-responsive cellulose nanofibril/sodium alginate (CNF/SA) hydrogel for drug release. The pH sensitivity of this hydrogel arises from the protonation and deprotonation of calcium alginate. Under acidic conditions (pH 1.5), alginate is protonated into an insoluble form, resulting in a low swelling ratio and slow drug release; under neutral or alkaline conditions (pH 7.4 and 11.0), alginate becomes deprotonated, leading to weakened hydrogen bonds between molecular chains and enhanced electrostatic repulsion, which causes significant swelling of the hydrogel and accelerates the release of ibuprofen (IBU). Ghauri et al. [61] successfully constructed a pH-sensitive hydrogel based on chitosan, sodium alginate, and polyethylene glycol (PEG) for controlled release of ceftriaxone sodium (CTX). In an acidic environment, protonation of the amino groups of chitosan generates electrostatic repulsion, causing the matrix to swell rapidly and resulting in fast drug release; in neutral or weakly alkaline environments, ionic interactions and hydrogen bonds between the polymer chains form a stable crosslinked network, allowing the drug to achieve slow, controlled release via Fickian or non-Fickian diffusion mechanisms, with the release process not altering the chemical stability of the drug.

The core advantages of pH-responsive release systems lie in their exceptional environmental adaptability and spatiotemporal controllability. By leveraging pH differences between physiological and pathological states, they enable site-specific triggering and on-demand drug delivery at targeted locations (such as the colon or wound microenvironment), thereby significantly improving bioavailability and reducing systemic toxicity and side effects. However, these systems also present certain environmental sensitivity risks and safety challenges. The release behavior is susceptible to dynamic instability caused by complex pH fluctuations in vivo, and the long-term biosafety of certain responsive components (such as metal ions or crosslinking agents) as well as the potential premature degradation of the material in non-target areas still require rigorous consideration.

#### 3.3.3. Temperature-Responsive Release

Temperature-responsive release of sodium alginate-based composite hydrogels involves compounding sodium alginate with thermosensitive polymers or constructing a temperature-responsive mechanism by regulating system components to form an intelligent drug release system. The mechanism involves temperature changes triggering structural transitions in the hydrogel matrix, altering network compactness, viscosity, or stability, thereby modulating the release rate of encapsulated cargo [70]. Chen et al. [62] constructed a calcium alginate/poly(N-isopropylacrylamide) (CAPH) hydrogel that utilizes the lower critical solution temperature (LCST) property of PNIPAAm, undergoing hydrophobic contraction at 34 °C, near body temperature. This mechanism promotes rapid release of mupirocin loaded in the hydrogel while assisting wound contraction. Nizioł et al. [63] successfully fabricated a thermosensitive hydrogel loaded with the antibacterial agent Octenisept^®^ using 3D printing technology. The hydrogel was composed of a PNIPAAm precursor, sodium alginate, and methylcellulose. When the environmental temperature exceeded the LCST of PNIPAAm, the polymer network contracted, thereby accelerating the release of the antibacterial agent. The hydrogel exhibited a faster drug release rate at 37 °C compared to 20 °C and demonstrated potent antibacterial effects against a variety of common pathogenic bacteria. Cao et al. [64] developed a thermosensitive Pluronic F127/alginate (GSNO-PL/AL) hydrogel capable of releasing nitric oxide (NO). The hydrogel remained liquid at low temperatures but transitioned into a gel at physiological temperature; this phase transition property effectively controlled the sustained release of NO from S-nitrosoglutathione (GSNO). Abbasi et al. [65] prepared thermosensitive hydrogel films by combining the thermoresponsive polymer Pluronic F-127 with sodium alginate and PVA. The temperature-responsive controlled release behavior of this dressing primarily relied on the micellization and sol–gel phase transition of F-127. At specific temperatures, the hydrophilic PEO chains and the central hydrophobic PPO block of F-127 form highly tightly packed supramolecular micellar structures, making the internal network of the hydrogel denser and thereby restricting drug molecule movement by reducing the diffusion coefficient.

The core advantage of temperature-responsive release strategies lies in their intelligent “on-demand drug delivery” regulation capability. Utilizing local wound temperature as an endogenous trigger signal enables autonomous drug release without external intervention, simplifying clinical procedures while significantly improving the spatiotemporal precision of drug administration. The sol–gel phase transition endows the material with good injectability and conformal filling capacity for irregular wound surfaces, while the physical restriction of drug diffusion by the dense network in the gel state effectively extends the therapeutic window and reduces the need for frequent dressing changes. However, this thermosensitive strategy still has inherent shortcomings. LCST-type polymers such as PNIPAAm have a relatively narrow phase transition temperature range, and fluctuations in body temperature or local temperature changes caused by wound inflammation can easily lead to unstable drug release behavior. Relying solely on a single temperature stimulus makes it difficult to meet the differentiated drug release requirements at different stages of wound healing, preventing precise regulation of release kinetics. Furthermore, the burst release effect caused by polymer phase transition and contraction can lead to a sudden increase in local drug concentration, posing a potential risk of cytotoxicity.

## 4. Applications of Sodium Alginate-Based Composite Hydrogels in Wound Dressings

Based on the structural characteristics endowed by the above preparation methods and the resulting drug release mechanisms, sodium alginate-based composite hydrogels exhibit diverse biomedical applications at all stages of wound repair. In recent years, sodium alginate-based composite hydrogel dressings have been increasingly developed, with additional components potentially possessing anti-inflammatory, antibacterial, pro-angiogenic, and hemostatic functions. The core processes through which they exert their reparative functions include hemostasis, anti-inflammation, angiogenesis, and epithelial repair. During the hemostasis phase, it is necessary to rapidly activate endogenous or exogenous coagulation factors to accelerate thrombus formation and stop bleeding; in the inflammatory phase, effective inhibition of pathogenic bacterial proliferation and clearance of excessive local reactive oxygen species (ROS) at the wound site are required to reduce tissue damage caused by inflammation; upon entering the proliferative phase, it is necessary to promote the proliferation and migration of vascular endothelial cells to construct a new vascular network, while simultaneously driving the proliferation and migration of epithelial cells toward the wound center to achieve wound coverage and tissue repair. In summary, sodium alginate-based composite hydrogel dressings, through synergistic effects of multiple components, can precisely regulate the core physiological processes at each stage of wound healing, providing an efficient and physiologically relevant new approach for wound repair [71,72].

### 4.1. Antibacterial Hydrogel

**Figure 10:**
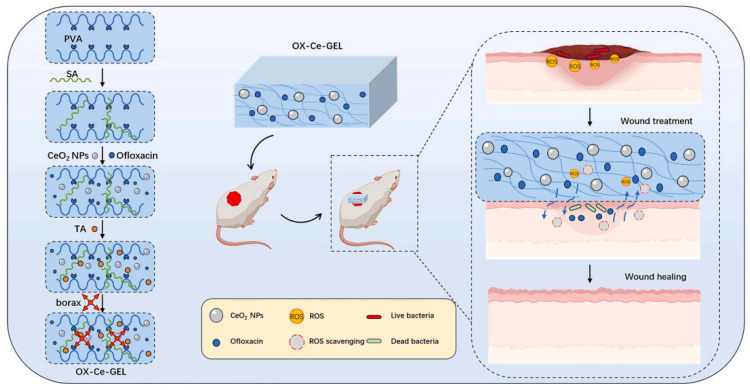
Synthetic scheme of hydrogel for diabetic wound healing [77]. Copyright 2024, Elsevier.

Bacterial infection is a major obstacle to wound repair. Therefore, hydrogels with antibacterial activity are a research focus for infectious wound healing. Their antibacterial effects primarily rely on surface contact killing or the release of loaded antibacterial agents. Among these, physical contact killing can be achieved through cationic groups on the hydrogel surface that disrupt bacterial membranes or through nanostructures that cause mechanical damage to bacteria [73]. Controlled release of antibacterial agents involves loading antibiotics, metal ions (such as silver, zinc, copper, etc.) [74,75]. Liu et al. [76] prepared a TA-GL/OSA/ZnO composite hydrogel dressing composed of gelatin, tannic acid, oxidized sodium alginate, and zinc oxide nanoparticles. The tannic acid and zinc oxide nanoparticles in the dressing exerted a synergistic antibacterial effect, achieving inhibition rates of 97.8% ± 0.9% against *S. aureus* and 96.6% ± 1.2% against *E. coli*. Hao et al. [77] loaded cerium dioxide nanoparticles and the antibacterial drug ofloxacin into a hydrogel to prepare a pH/glucose dual-responsive hydrogel, as shown in Figure 10. This hydrogel exhibited excellent antibacterial performance, with inhibition rates of 93.4% against *S. aureus* and 96.3% against *E. coli*. Its responsive release characteristics are well-suited to the low pH and high glucose microenvironment of diabetic wounds, with drug release at pH 5.5 being 67.8% higher than that at pH 7.4, providing a new approach for the development of chronic wound dressings. Rata et al. [78] used hyaluronic acid (HA) and sodium alginate (AG) as raw materials to form an anti-inflammatory and antibacterial composite hydrogel for burn wound treatment via an esterification reaction, loading the anti-inflammatory drug ibuprofen and zinc oxide nanoparticles (ZnO NPs). Experimental results showed that the hydrogel containing 5% ZnO NPs exhibited good antibacterial activity against *S. aureus*, with an inhibition zone diameter of 22–23 mm. The ibuprofen in the composite hydrogel achieved controlled release, with a drug release rate of 68–95% within 24 h, providing sustained analgesic effects. Xu et al. [79] developed a multifunctional smart hydrogel dressing named SPC. The SPC hydrogel demonstrated excellent antibacterial performance. Under light irradiation, the hydrogel achieved bactericidal efficiencies of up to 99.63% against methicillin-resistant Staphylococcus aureus (MRSA) and 98.95% against extended-spectrum β-lactamase-producing *E. coli*. Meanwhile, it exhibited a strong inhibitory effect on MRSA biofilms, with an inhibition rate of 94.3%. This high-efficiency antibacterial effect is primarily attributed to the hydrophobic microenvironment formed by PNIPAM in the hydrogel, which promotes the aggregation of the coumarin-based photosensitizer (C3AC), effectively killing bacteria and disrupting the biofilm structure. Aljayyousi et al. [80] prepared a hydrogel based on PVA/gelatin/sodium alginate loaded with Ceragenin CSA-44 for burn wound treatment. In terms of antibacterial activity, the hydrogel showed rapid inhibitory effects against *E. coli*, Pseudomonas aeruginosa, *S. aureus*, and MRSA, completely suppressing these bacteria within 2 h, 2.5 h, 3 h, and 3.5 h, respectively. Yuan et al. [81] developed a bacteriophage-loaded aldehyde-modified sodium alginate/gelatin/carboxymethyl chitosan/phage hydrogel 3 (AGCP3) for the treatment of wounds infected with multidrug-resistant bacteria. In terms of antibacterial activity, the AGCP3 hydrogel loaded with bacteriophage killed 100% of MRSA within approximately 12 h and almost completely eliminated bacterial biofilms within 24 h. In in vivo experiments, the AGCP3 hydrogel significantly accelerated the healing of MRSA-infected wounds, achieving a wound closure rate of 96% after 14 days. Existing research on sodium alginate-based composite hydrogels has successfully developed dressings with high-efficiency antibacterial and biofilm-inhibiting effects against *S. aureus, E. coli*, and multidrug-resistant bacteria (such as MRSA) by integrating various strategies including nanoparticles, antibacterial drugs, photosensitizers, antimicrobial peptides, and bacteriophages. Some studies have also achieved intelligent responsive release tailored to the diabetic wound microenvironment and demonstrated in vivo effects in promoting wound healing. Future efforts should continue to focus on further enhancing the stability of long-term antibacterial effects, ensuring material biosafety, optimizing preparation processes, and actively promoting its practical application in the treatment of clinically infected wounds. In particular, addressing the challenge of bacterial drug resistance, as well as ensuring effective local concentrations of antibacterial agents at the wound site without inducing cytotoxicity, remain key issues to be urgently resolved in future research.

### 4.2. Anti-Inflammatory Hydrogel

Anti-inflammatory hydrogels are a class of multifunctional biomedical materials that can exert wound anti-inflammatory repair effects through multiple pathways. They can both serve as physical barriers to isolate external stimuli and load active components such as curcumin and non-steroidal anti-inflammatory drugs for controlled drug release [82]. At the same time, they can scavenge excessive reactive oxygen species (ROS) at the wound site, selectively capture inflammatory factors, and regulate the immune microenvironment, inducing the polarization of macrophages from the pro-inflammatory M1 phenotype to the reparative M2 phenotype [83]. Wei et al. [84] prepared an Eu^3+^-mediated double-crosslinked collagen mimetic peptide/sodium alginate composite hydrogel (SA-E-D-C) with excellent mechanical strength (maximum compressive stress of 13.8 kPa), 3D printability, self-healing ability, and controlled sustained release properties. In terms of anti-inflammatory effects, it effectively scavenged ROS, significantly reducing ROS levels in human adult low-calcium high-temperature keratinocytes (HaCaT cells), inhibited the expression of pro-inflammatory markers inducible nitric oxide synthase (iNOS) and interleukin-6 (IL-6), and promoted M2-type macrophage polarization, thereby alleviating wound inflammation. Song et al. [85] developed an injectable tannic acid@alginate/boron-containing bioactive glass/calcium ion hydrogel (TA@SA/BG/Ca^2+^) multifunctional hydrogel with enhanced mechanical properties (storage modulus and loss modulus one order of magnitude higher), adhesiveness, high water content, and staged ion release capability. In terms of anti-inflammatory effects, the B^3+^, Si^4+^, and Ca^2+^ ions released from the hydrogel synergistically inhibited bacterial growth and inflammation, promoted macrophage polarization toward the M2 phenotype, and transcriptomic analysis showed significant downregulation of inflammatory mediators and activation of the phosphatidylinositol 3-kinase/protein kinase (PI3K-Akt) signaling pathway, thereby effectively reducing the inflammatory response in infected wounds. Tao et al. [86] prepared a curcumin-Co-ZIF-8@oxidized sodium alginate/carboxymethyl chitosan/tannic acid hydrogel (CCZ@OSA/CMCS/TA) with excellent mechanical properties (fracture stress of 132 kPa), self-healing ability, and pH-responsive release characteristics. In terms of anti-inflammatory effects, the hydrogel demonstrated strong ROS scavenging capacity, significantly downregulated the mRNA expression of the pro-inflammatory cytokines tumor necrosis factor-α (TNF-α) and interleukin-1β (IL-1β) in M1-type macrophages, and promoted the transition of macrophages from M1 to M2 phenotype, thereby effectively inhibiting inflammation. Zhang et al. [87] prepared carbon quantum dots (CDs) from *Scutellaria baicalensis* leaves and incorporated them together with baicalein (BAI) into a sodium alginate hydrogel to form a baicalein-carbon dots/sodium alginate hydrogel (BCG), which possessed a stable gel network, high water absorption capacity, biodegradability, and sustained release properties. In terms of anti-inflammatory effects, the BCG hydrogel effectively scavenged ROS (H_2_O_2_ scavenging rate of 81.19%, Superoxide anion (O_2_^−^) scavenging rate of 67.46%, DPPH• scavenging rate of 88.49%), significantly reduced ROS, CD86, and iNOS levels in the wound, activated the nuclear factor erythroid 2-related factor 2 (Nrf2) antioxidant signaling pathway, and inhibited the nuclear factor-kappa B (NF-κB) inflammatory signaling pathway, thereby alleviating early wound inflammation. He et al. [88] prepared a gelatin/sodium alginate double-network hydrogel (CEGA) loaded with antler stem cell conditioned medium (ASC-CM) and epigallocatechin gallate (EGCG), which exhibited excellent swelling capacity (up to 900%), sustained release properties, and good mechanical strength and biocompatibility. In terms of anti-inflammatory effects, the CEGA hydrogel effectively scavenged ROS, significantly reducing the mean fluorescence intensity in the H_2_O_2_ + CEGA group, promoted the expression of the anti-inflammatory factor IL-10 while inhibiting the expression of the pro-inflammatory factor TNF-α, and, most importantly, effectively promoted macrophage polarization from M1 to M2 phenotype, thereby alleviating the inflammatory response in diabetic wounds. Ruan et al. [89] used sodium alginate (AL) and Pluronic F127 (PL) as a matrix to form a PL/AL gel via low-temperature dissolution, then added BA camellia oil submicron emulsions (BA/COs) and S-nitrosoglutathione (GSNO) under light-protected stirring to prepare a BA/COs/NO-PL/AL thermosensitive hydrogel for wound healing. This hydrogel inhibited IL-6 production in RAW264.7 cells, downregulated the expression of TNF-α, IL-6, and IL-1β in the wound, and promoted epithelial tissue formation and collagen deposition at the wound site, thereby accelerating healing. To improve the excessive inflammatory microenvironment of wounds and alleviate oxidative stress damage, multiple studies have used sodium alginate as a substrate and incorporated various functional components to construct a series of highly efficient anti-inflammatory hydrogel dressings. These materials primarily function by efficiently scavenging wound ROS, alleviating oxidative stress damage, downregulating the expression of pro-inflammatory cytokines such as TNF-α, IL-6, and IL-1β, and regulating the polarization of macrophages from the M1 pro-inflammatory phenotype to the M2 reparative phenotype, thereby effectively improving the wound inflammatory microenvironment and adapting to various inflammation repair scenarios, including ordinary trauma and diabetic wounds. Future research focuses on efficient scavenging of reactive oxygen species, precise regulation of macrophage polarization, and alleviation of inflammation at the molecular level by activating signaling pathways such as PI3K-Akt and Nrf2 while inhibiting the NF-κB pathway, thereby promoting wound healing and angiogenesis, ultimately achieving personalized and more effective wound dressing applications.

### 4.3. Hemostatic Hydrogel

Hemostatic hydrogels can rapidly adsorb wound exudates, maintain a moist microenvironment, and interact with blood components, exhibiting significant hemostatic performance. The calcium ions in crosslinked sodium alginate hydrogels can activate platelets and induce fibrin generation, thereby accelerating the coagulation process [90]. Liu et al. [91] prepared an OSA/CMC composite hemostatic hydrogel via Schiff base reaction. Experiments showed that its coagulation time decreased significantly with increasing oxidation degree, dropping from approximately 9 min in the control group to 2.44 ± 0.52 min. In mouse tail amputation and liver hemorrhage models, the hydrogel with 10% oxidation degree also demonstrated excellent hemostatic effects, greatly shortening hemostasis time and significantly reducing blood loss, confirming that OSA/CMC hydrogels possess characteristics such as rapid gelation, high coagulation efficiency, and excellent in vitro and in vivo hemostatic performance. Chen et al. [92] prepared SACC-GO hemostatic powder via a simple ball milling method, which could rapidly form a gel upon contact with blood, as shown in the figure. In vitro experiments showed that pure SA powder could not crosslink into a gel or promote coagulation, whereas SACC-GO, SAC, and SACC all crosslinked with blood within 1 min to form a gel, achieving rapid hemostasis. In in vivo experiments using a rat tail injury model, the average blood loss in the SACC-GO group was 352 mg, far superior to the 1487 mg in the control group. In a femoral artery massive hemorrhage model, the hemostasis time was reduced to 47.1% of the control group, and blood loss was reduced to 32.0%; in a liver trauma model, hemostasis time was also significantly shortened and blood loss reduced, highlighting its efficient hemostatic performance for venous, arterial, and visceral bleeding. Wu et al. [93] prepared a double-network composite hydrogel using carboxymethyl chitosan, sodium alginate, and methyl alginate as raw materials via covalent crosslinking and electrostatic interactions. The hydrogel exhibited rapid blood absorption and hemostatic performance superior to gauze and commercial gelatin sponges. In rat liver hemostasis experiments, coagulation time was significantly shortened. In whole blood coagulation tests, its deionized water rinse was almost colorless, indicating stronger coagulation ability. Moreover, its good biocompatibility laid the foundation for subsequent wound vascular regeneration, warranting further investigation. Lu et al. [94] loaded Jianpi Huayu essential oil (JEO) into nanoparticles (ZCJ) and embedded them in a hydroxypropyl methylcellulose/Pluronic F-127/sodium alginate (HPS) hydrogel matrix to prepare a nanocomposite hydrogel (HPS@ZCJ) for hemostasis and prevention of postoperative recurrence in liver cancer surgery. The in vitro hemostatic effect of this hydrogel was comparable to that of fibrin gel, achieving a blood coagulation index (BCI) of 28.13 ± 10.62% at 5 min and an erythrocyte adhesion rate of 48.64 ± 2.29%. In a rat non-compressive liver hemorrhage model, the blood loss was only 253.5 ± 67.23 mg, significantly lower than the 920.7 ± 244.19 mg in the PBS group. Yuwei Zhou et al. [95] constructed a multiphase injectable hydrogel (MH) with a combined microstructure for wound hemostasis under dynamic mechanical environments. The study found that this hydrogel exhibited excellent hemostatic performance. In a liver injury model, the injected MH rapidly covered the wound and achieved almost immediate hemostasis, reducing blood loss by 86.38 ± 2.48% compared to the control group, significantly outperforming the chitosan hemostatic powder (CHP) group and the alginate dressing (AD) group, with a shorter hemostasis time. In a femoral artery hemostasis model, the MH group showed a blood loss reduction of 66.19 ± 4.21%, also superior to the CHP and AD groups. This study provides an effective strategy for constructing hemostatic injectable hydrogels suitable for dynamic mechanical environments. Zhang et al. [96] prepared a nanoscale zero-valent iron-driven sodium alginate/polyacrylic acid composite hydrogel (SPI), which was dried and ground to obtain SPI_6_ powder. This powder exhibited strong blood absorption capacity and self-gelation properties, inducing whole blood coagulation within 30 s in vitro, with coagulation efficiency superior to that of commercial chitosan hemostatic powder (CCS). In a rat liver injury model, the blood loss within 3 min was only 36 mg, significantly lower than the 284 mg in the control group and the 113 mg in the CCS group, achieving hemostasis within 30 s without blood penetration. Furthermore, in various acute bleeding models including the heart, femoral artery, and tail vein, rapid hemostasis was achieved within 30 s, with no rebleeding after 10 min, demonstrating stable and reliable hemostatic efficacy. In the wound repair process, rapid and effective hemostasis is the primary prerequisite for controlling wound injury and ensuring subsequent healing. To this end, existing research has prepared a variety of hemostatic hydrogels and hemostatic powder materials using sodium alginate as a substrate. These materials, usually optimized via composite modification, possess rapid gel formation, strong pro-coagulant, and blood absorption properties. In both in vitro and in vivo experiments, they significantly shorten coagulation time and greatly reduce wound blood loss, with hemostatic effects superior to traditional gauze and commercial hemostatic products, demonstrating satisfactory hemostatic performance for acute venous, arterial, and visceral bleeding scenarios. Future efforts need to focus on optimizing material formation speed and wound fit performance, improving hemostatic stability under strong blood flow impact and long-term anti-rebleeding capability, and perfecting hemostatic validation studies in complex trauma and large animal models in vivo, continuously promoting the translation of sodium alginate-based hemostatic materials from fundamental laboratory research to large-scale clinical applications in emergency hemostasis and surgical intraoperative hemostasis.

### 4.4. Pro-Angiogenic Hydrogel

**Figure 11:**
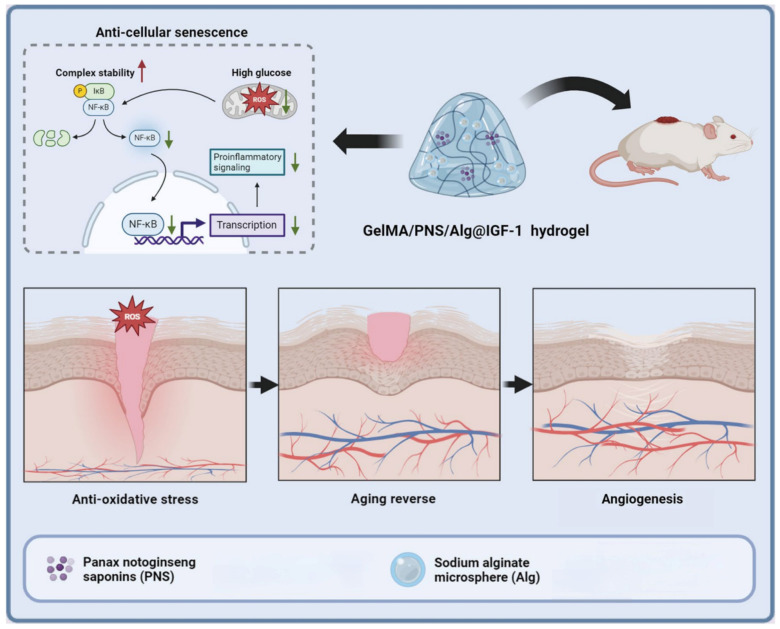
Repair of diabetic skin wounds, total saponins from *Panax notoginseng*, insulin-like IGF-1, methacrylated gelatin hydrogel, and alginate-based microspheres [99]. Copyright 2025, Springer Nature.

Pro-angiogenic hydrogels have a chemical composition similar to the natural extracellular matrix, are rich in bioactive molecules, possess good biocompatibility, and promote cell proliferation and differentiation while avoiding immune reactions. Furthermore, the appropriate porosity of the hydrogel facilitates the sustained release of growth factors, thereby promoting the formation of capillary networks [97]. Jabeen et al. [98] prepared *N*-(3-trimethoxysilylpropyl)ethylenediamine crosslinked sodium alginate hydrogels with the addition of different concentrations of polyethylene glycol (PEG). In the chick chorioallantoic membrane (CAM) assay, hydrogels containing PEG significantly increased the number of blood vessels, with the ANP2 group exhibiting the highest vessel count of 25.05 ± 0.0513, compared to 13.02 ± 0.3600 in the control group (*p* ≤ 0.05). Histological analysis confirmed that the hydrogel could enhance re-epithelialization, indicating that it possesses prominent effects in promoting angiogenesis and epithelialization. Yang et al. [99] developed a multifunctional hydrogel (GelMA/PNS/Alg@IGF-1) that integrated gelatin methacryloyl (GelMA), *Panax notoginseng saponins* (PNS), and insulin-like growth factor-1 (IGF-1) encapsulated in alginate microspheres. Under high-glucose conditions in vitro, this hydrogel significantly promoted the migration and tube formation of human umbilical vein endothelial cells (HUVECs) and resulted in a clearer vascular network. In an in vivo diabetic wound model, the hydrogel significantly enhanced CD31-labeled neovascularization, and accelerated granulation tissue formation and re-epithelialization. Mao et al. [100] prepared a novel sodium alginate–arginine–zinc ion (SA-Arg-Zn^2+^) hydrogel membrane, as shown in Figure 11. This hydrogel promoted angiogenesis by upregulating VEGF expression. ELISA test results showed that VEGF expression in the SA-Arg-Zn^2+^ hydrogel membrane group was significantly higher than that in the control group, and H&E staining also confirmed accelerated angiogenesis. This hydrogel film can also promote fibroblast proliferation and remarkably accelerate skin wound healing, achieving a 100% wound closure rate on day 14, which is closely associated with enhanced re-epithelialization. Chi et al. [101] prepared a dopamine-modified oxidized sodium alginate hydrogel (OSA-DA). In in vitro experiments, the OSA-DA hydrogel significantly promoted HUVEC migration and tube formation, with the tube formation numbers in the OSA-DA1 and OSA-DA2 groups being 1.25-fold and 1.33-fold higher than the control group, respectively. In a chronic wound model in diabetic rats, the OSA-DA hydrogel significantly promoted angiogenesis by increasing the expression of CD34 and VEGF. In addition, the hydrogel can facilitate epidermal tissue regeneration, thereby accelerating wound healing. Xu et al. [102] prepared an injectable bioactive glass/sodium alginate (BG/SA) hydrogel. In in vivo experiments, this hydrogel significantly promoted angiogenesis during tendon healing. Immunohistochemical staining showed that the BG/SA group exhibited significantly more CD31-positive signals and circular vascular structures than the control group, and the number of mature blood vessels co-stained with CD31/α-SMA was also significantly increased, indicating a marked promotion of capillary and mature blood vessel formation. Zheng et al. [103] developed an ROS-responsive liposome composite hydrogel encapsulating the mitochondrial-targeted antioxidant SS-31 and the pro-angiogenic molecule S1P. In a rat model of myocardial infarction, this hydrogel significantly promoted endothelial cell angiogenesis by releasing S1P and activating the S1PR1/PI3K/Akt signaling pathway. CD31 immunofluorescence staining showed that the hydrogel treatment groups loaded with S1P/Lipo or S1P/SS-31/Lipo exhibited significantly higher vascular density in the infarct region than other formulation groups. Effective neovascularization in the later stage of wound repair is a key core link in wound tissue repair and healing. To this end, existing research has constructed a variety of composite hydrogel systems with pro-angiogenic activity using sodium alginate as a matrix. These hydrogels can effectively promote endothelial cell migration and tubular structure formation, significantly upregulate the expression of angiogenesis-related genes and proteins such as VEGF, CD31, and CD34, and continuously induce neovascularization and maturation by activating relevant signaling pathways. They have been shown to significantly increase vascular density and accelerate vascular network reconstruction in various models including ordinary trauma, diabetic chronic wounds, and myocardial injury. In addition, these materials can regulate the migration, proliferation, and collagen deposition of keratinocytes and fibroblasts, synergistically promoting the epithelialization process, accelerating wound closure, and reducing scar formation. Future research needs to further precisely regulate the temporal release rhythm of pro-angiogenic factors, balance the processes of angiogenesis and tissue remodeling, and promote the clinical translation and application of sodium alginate-based pro-angiogenic hydrogels in the repair of various chronic and hard-to-heal wounds.

## 5. Conclusions and Challenges

This article systematically reviews the research progress of sodium alginate-based composite hydrogels in the field of wound dressings. Leveraging their excellent biocompatibility and the tunability of their structure and properties, sodium alginate-based composite hydrogels have become key materials for addressing the pain points of traditional wound dressings, such as insufficient antibacterial activity, easy adhesion, and poor adaptability. Their preparation methods, involving the synergistic development of physical and chemical crosslinking, overcome single limitations and precisely regulate the hydrogel network structure and crosslinking density, laying the core technical foundation for the directional optimization of their properties. Drug release mechanisms have evolved from basic sustained release to precise controlled release and pH/temperature/light-responsive triggered release, demonstrating enormous application potential in wound repair scenarios such as anti-inflammation, antibacterial action, rapid hemostasis, and pro-angiogenesis, providing new ideas for the treatment of acute wounds, chronic infected wounds, and diabetic foot ulcers. Overall, current research in this field still faces multiple common challenges, as detailed below:

(1) Immature large-scale preparation processes and high costs: Current fabrication mainly relies on laboratory-scale conditions, leading to poor batch stability and reproducibility. Ionic and chemical crosslinking techniques are difficult to scale up, while some components are expensive. Low-cost, continuous green manufacturing routes are lacking, making it hard to meet the demands of industrial mass production and clinical popularization.

(2) Prominent difficulties in regulatory approval and clinical translation: Hydrogel dressings are classified as Class III medical devices, requiring strict compliance with regulatory standards for biocompatibility, degradation behavior, and in vivo residue. Current research remains mostly limited to in vitro and animal experiments, lacking sufficient data on long-term in vivo toxicity, immunogenicity, and histocompatibility. As a naturally derived polymer, sodium alginate has inherent batch-to-batch differences in purity, molecular weight distribution, mechanical integrity and structural stability, making full standardization difficult. Establishing unified quality control specifications for natural polysaccharide raw materials is an essential regulatory threshold for the commercial application of sodium alginate-based hydrogel dressings. In addition, the absence of unified industrial quality standards and quality control systems leads to long approval cycles and high barriers to clinical transformation.

(3) Insufficient long-term in vivo safety and stability: Physically crosslinked hydrogels tend to dissociate and lose mechanical strength under physiological conditions. Residual chemical crosslinkers may trigger inflammation or cytotoxicity. The long-term accumulation, potential toxicity and metabolic pathways of composite components remain unclear. The mismatch between material degradation rate and tissue regeneration can easily lead to foreign body reactions or delayed healing.

(4) Insufficient intelligent response design and functional integration: Existing hydrogels are mostly single-responsive and cannot precisely match the dynamic changes of wound microenvironments involving pH, reactive oxygen species (ROS), and enzymes. They lack temporally controlled and multi-signal synergistic release mechanisms. Advanced manufacturing technologies such as 3D/4D printing are not sufficiently integrated with hydrogels, making it difficult to achieve personalized, complex wound-adapted and structure–function integrated designs, thus limiting their precise repair capabilities.

## 6. Future Perspectives

In response to the above challenges, future research should be guided by industrialization, clinical translation and intelligence, focusing on four key directions: large-scale preparation, regulatory compliance, long-term safety and intelligent design, to promote the transformation of sodium alginate-based hydrogels from basic research to clinical applications and industrialization.

(1) Develop low-cost, scalable green preparation technologies: Optimize ionic crosslinking, Schiff base crosslinking and other processes to simplify procedures and reduce energy consumption; develop continuous gelation, in situ crosslinking and injectable molding technologies to improve batch stability; adopt biomass substitution to reduce the cost of functional components; and establish quality control standards and online monitoring systems to lay the foundation for industrial mass production.

(2) Improve long-term in vivo safety evaluation and regulatory compliance pathways: Systematically conduct studies on long-term toxicity, chronic inflammation, immunogenicity, and in vivo degradation kinetics; focus on evaluating residual crosslinkers, nanoparticle accumulation, and off-target effects of active factors; establish a four-level safety evaluation system covering materials, cells, tissues, and organs; align with medical device regulatory requirements; unify quality control indicators; and formulate preclinical evaluation specifications to shorten approval cycles and reduce translation risks.

(3) Develop multi-responsive intelligent hydrogels and integrate advanced manufacturing systems: Design hydrogels with synergistic responses to pH, temperature, reactive oxygen species (ROS) and enzymes, to precisely match the dynamic wound microenvironment; develop temporally controlled release systems to achieve programmed repair: antibacterial and anti-inflammatory first, followed by angiogenesis, and finally epithelialization; integrate 3D/4D printing, in situ forming and electrospinning technologies to fabricate personalized, porous-gradient, structure–function integrated dressings that adapt to irregular, deep and dynamic wounds; and combine biosensing and visual monitoring to enable real-time feedback and precise regulation of wound repair.

(4) Promote industry–university–research–medical collaboration and clinical translation: Strengthen interdisciplinary integration of materials science, biology, medicine and engineering; collaborate with enterprises, hospitals and regulatory agencies, focusing on high-value and unmet clinical needs; conduct large animal model studies and multicenter clinical trials to verify efficacy and safety; advance product registration, standard formulation and industrialization; and ultimately, achieve large-scale application of sodium alginate-based intelligent hydrogel dressings from laboratory to clinic, providing an efficient, safe and precise new generation therapy for wound repair.

## Figures and Tables

**Table 1:** Physical crosslinking methods and their advantages and disadvantages.

Crosslinking Method	Advantages	Disadvantages	References
Ionic Crosslinking	Simple operation Rapid gelation Easy to regulate Good antibacterial activity and biocompatibility	Weak ionic bonds Poor structural stability Prone to ion-induced dissociation Weak single-crosslink mechanical property	[4,5,6,7]
Hydrogen Bond Crosslinking	High tensile strength Superior self-healing Biocompatibility Synergistic with other crosslinking methods	Poor mechanical strength Sensitive to pH, temperature and ions Unstable in single hydrogen crosslinking	[8,9,10,11]
Hydrophobic Interaction	Flexible regulation, rapid gelation, high structural stability, and encapsulation of lipophilic actives	Crosslinking hard to regulate Temperature-sensitive stability Partial chemical modification required	[12,13,14,15]
Electrostatic Interaction	Sensitive pH responsiveness Flexible regulation, fast gelation, stable structure	Vulnerable to ionic disruption Weak mechanical strength Poor long-term in vivo stability	[16,17,18,19]

**Table 2:** Chemical crosslinking methods and their advantages and disadvantages.

Crosslinking Method	Advantages	Disadvantages	References
Schiff base crosslinking	Strong adhesion Dynamic reversibility pH-responsive self-healing capability	Poor swelling capacity System may induce flocculation Insufficiently validated long-term in vivo performance	[25,26,27,28]
Photocrosslinking	High drug loading and controlled release, excellent anticoagulation, good functional extensibility and fast crosslinking	Weak sustained release Hard crosslinking density regulation	[29,30,31,32]
Radiation crosslinking	Good light transmittance Stable mechanical strength Superior water absorption and retention	High cost Complex process parameters Difficulty in regulating radiation dose	[33,34,35,36]
Enzymatic crosslinking	Mild reaction conditions Catalytic specificity Stable structure Good biocompatibility	Environmentally sensitive Potential incomplete drug release Extremely slow gelation rate when used alone	[37,38,39,40]

**Table 3:** Characteristics, advantages and limitations of drug release mechanisms.

Release Mechanism	Advantages	Disadvantages	References
Sustained Release	No obvious burst release; Good biocompatibility	Poor efficacy of single matrix; Uneven local drug release; Insufficient stability of release rate	[46,47,48,49]
Controlled Release	Controllable drug release; Excellent targeting ability; Sensitive responsiveness	Hard to balance targeting and stability; In vivo interference on drug release	[50,51,52,53]
Photo-responsive Release	Precise response; Diversified functions; On-demand drug release achievable	Limitation of light irradiation; Potential phototoxicity risk; Complicated process and high cost	[54,55,56,57]
pH-responsive Release	Stable drug release; Strong targeting ability; Adaptable to in vivo pH differences	Poor stability; In vivo pH fluctuation triggers uncontrolled drug release	[58,59,60,61]
Thermo-responsive Release	Efficient drug release; High temperature sensitivity; Prolonged drug retention time	Low adaptability; Hard gelation temperature control; Temperature-dependent stability	[62,63,64,65]

## Data Availability

No new data were created or analyzed in this study. Data sharing is not applicable to this article.
